# The abscopal effect of anti-CD95 and radiotherapy in melanoma

**DOI:** 10.1007/s12672-023-00682-7

**Published:** 2023-05-16

**Authors:** Jixiang Xu, JiangFeng He, JiaJun He, Yuanmin He, DaoJun Zhang, Rui Kong, Kena Dan

**Affiliations:** 1 Department of Dermatology, The Affiliated Hospital of Southwest Medical University, Zhongshan 319, Luzhou, 646000 China; 2 Nursing Department, The Affiliated Hospital of Southwest Medical University, Zhongshan 319, Luzhou, 646000 China; 3 Department of Dermatology, The Third Affiliated Hospital of ChongQing Medical University, ShuangHuZhiLu 1, Chongqing, 401120 China; 4 Department of Oncology, The Third Affiliated Hospital of ChongQing Medical University, ShuangHuZhiLu 1, Chongqing, 401120 China

**Keywords:** anti-CD95, Immune response, Melanoma, Radiation

## Abstract

**Background:**

Radiotherapy (RT) is frequently adopted to control cancer cell proliferation, which is achieved by altering the tumor microenvironment (TME) and immunogenicity. Apoptosis of cancer cells is the major effect of radiation on tumor tissues. Fas/APO-1(CD95) receptors on the cell membrane are death receptors that can be activated by diverse factors, including radiation and integration with CD95L on CD8^+^ T cells. The abscopal effect is defined as tumor regression out of the local RT field, and it is produced through anti-tumor immunity. The immune response against the radiated tumor is characterized by the cross-presentation between antigen-presenting cells (APCs), which includes cytotoxic T cells (CTLs) and dendritic cells (DCs).

**Methods:**

The effect of activation and radiation of CD95 receptors on melanoma cell lines was examined in vivo and in vitro. In vivo, bilateral lower limbs were given a subcutaneous injection of a dual-tumor. Tumors in the right limb were radiated with a single dose of 10 Gy (primary tumor), while tumors in the left limb (secondary tumor) were spared.

**Results:**

The anti-CD95 treatment plus radiation (combination treatment) reduced growth rates of both primary and secondary tumors relative to the control or radiation groups. In addition, higher degrees of infiltrating CTLs and DCs were detected in the combination treatment compared to the other groups, but the immune response responsible for secondary tumor rejection was not proven to be tumor specific. In vitro, combination treatment combined with radiation resulted in further apoptosis of melanoma cells relative to controls or cells treated with radiation.

**Conclusions:**

Targeting CD95 on cancer cells will induce tumor control and the abscopal effect.

**Supplementary Information:**

The online version contains supplementary material available at 10.1007/s12672-023-00682-7.

## Introduction

The abscopal effect occurs with radiation treatment [1]. It not only shrinks the targeted tumor but also leads to shrinkage of untreated tumors elsewhere in the body. Although the underlying mechanisms responsible for the abscopal effect are still being investigated, the immune system is thought to play an important role [2–8]. Local radiotherapy (RT) can provide immunogenic cell death (ICD) to produce several internal damage-associated molecular patterns (DAMPs), including adenosine triphosphate (ATP) and high-mobility group box-1 protein (HMGB1), which play key roles in priming the immune system through stimulating dendritic cells (DCs), thereby giving rise to enhanced antigen presentation to T cells [4, 9]. Furthermore, radiation increases the levels of NKG2D ligands, CD95 (Fas), and major histocompatibility complex (MHC1) in cancer cells, which are finally eliminated by natural killer (NK) cells and T cells [8, 10–13]. However, to attain the most benefits from RT, standard chemotherapeutics should be used in combination with immune modulators or radiation-sensitizing agents. Several biotherapies will affect the immune system and cause target cell death, called apoptosis.

Apoptosis can be induced by two different mechanisms, which are referred to as extrinsic and intrinsic pathways. The extrinsic apoptosis pathway is activated by transmembrane receptor interactions, which are death receptors and belong to the tumor necrosis factor (TNF) superfamily. These receptors encompass CD95 (Fas), TNFR1, DR3, DR4, and DR5. Binding CD95L with CD95 results in recruitment of the adapter protein FADD, which then combines with procaspase-8. During this, a death-inducing signalling complex (DISC) is produced and procaspase-8 is catalysed. The activated caspase 8 executes the apoptosis process [14, 15]. CD95 is expressed by many tumor cell lines and its physiological ligand is CD95L. CD95L is expressed by T lymphocytes and natural killer cells during an active immune response [16, 17]. It is known that ligation of CD95 constitutes one of the two major actions of the lymphocytes to mediate an immune response; the other is the release of the cytolytic molecules perforin and granzymes in the targeted cells. So far more and more studies confirmed that It was unlikely that the function of CD95 was only to induce apoptosis [18]. CD95 involved activation of NF-κB and the three MAP kinases, Erk1/2, JNK1/2, and p38, thereby related with tumor-promoting activities [19–21]. Besides, Teodorczyk et al. found out that CD95-mediated activation of Sck/Shc2 is indispensable for cell cycle progression of metastatic pancreatic ductal adenocarcinoma [22]. Kleber, Susanne, et al. study also confirm that CD95 mediates invasion via the Src/PI3K/GSK3*β*/MMP (matrix metalloproteinase) pathway [23]. The concept that CD95 could be a tumor promoter has gained wide acceptance and inhibiting CD95 could be a new therapeutic strategy to cancers.

In this study, we investigated the effect of CD95 agonists in combination with radiation on stimulating the abscopal effect. B16F10 and YUMM 1.7 tumor cells were injected simultaneously into the lower limbs of C57BL/6 mice, and subsequently, mice were exposed to radiation alone, or CD95 agonists alone or both. The abscopal effect was observed in mice receiving combination therapy at 6 days’ post-radiation.

## Materials and methods

### Cell lines, mice, and reagents

B16F10, YUMM 1.7 and A375-MA1 tumor cell lines were obtained from the American Type Culture Collection (Manassas, VA, USA) and were grown at 37 °C in 5% CO_2_ in Dulbecco’s Modified Eagle’s Medium (DMEM) with 10% fetal bovine serum plus 1% glutamine-penicillin-streptomycin.

Z-VAD-fmk (627610) was purchased from Merck (Darmstadt, Germany). CD95 Polyclonal Antibody (115214) was from Thermo Fisher Scientific. Purified Hamster anti-Mouse CD95/JO2 (554255) was from BD Biosciences. Purified anti-CD16/CD32 (101301), anti-CD45 (103101), anti-CD8 (100701), anti-CD11b (101207), and anti-CD11C (117301) antibodies were from Biolegend.

### Mouse model

The mice model has been described in previous studies[3, 24]. C57BL/6 mice (8-10-week-old) were used. Mice were caged in groups of five or less, and their hind limbs were shaved. 1 × 10^5^ B16F10 and YUMM 1.7 cells were implanted in the right limbs of mice (Primary tumor), and after 2 days cells were implanted in the left lower limbs (Secondary tumor). The tumor volume was calculated according to the following formula: Tumor size=(length)*(width)^2^/2. Two weeks after tumor implantation the tumor reached a volume about 100 mm^3^ at the initiation of treatment. Maximum tumor volume in our experiment is not exceed 2000 mm^3^ for mice. Mice were euthanized when they have near maximum tumor volume or serious adverse reactions, such as weight loss, abnormal behavior, severe skin damage, etc. Animal care and experimental procedures were conducted according to Institutional Animal Care and Use guidelines. Animal experiments were approved by the Institutional Animal Care and Treatment Committee of Southwest Medical University (SWMU2019083712) (Luzhou, China).

### Colony-forming assay and in vitro experiments

Cells were harvested by removing the medium, washing the cells with PBS, and using trypsinization to produce single-cell suspensions. A sufficient volume of medium supplemented with serum was added and the cells were counted. The cells were diluted to a density of 100 cells/ml and 2 ml was added to each well of 6 well plates. After the cells were attached, after about 2 h, cells were either left without treatment, treated with anti-CD95, radiated, or treated with a combination of CD95 and radiation. After 7 days, the medium was removed and the cells were rinsed with PBS before adding 2 ml of 0.5% crystal violet for 30 min. The crystal violet was removed, and the cells were rinsed and left in RT, and colonies were counted.

### MTT viability assay

Cells (3 × 10^5^/well) were inoculated in 24-well plates and cultured for a period of 24 h. Next, cells were treated with 2 Gy radiation alone, 100 ng/mL anti-CD95 alone, 2 Gy + anti-CD95, Z-VAD alone, or Z-VAD + 2 Gy + anti-CD95, followed by incubation for 24, 48 and 72 h, respectively. DMSO alone (vehicle)-treated cells served as controls. According to previous experiments, DMSO had no effect on cell proliferation. The MTT assay was performed to determine cell viability following respective treatment according to a previous study [25]. The EnSpire Multimode Plate reader (PerkinElmer, Milano, Italy) was utilized to measure absorbance at 550 nm.

### Tumor radiation and administration of anti-CD95

Purified Hamster anti-Mouse CD95/JO2 was diluted in PBS as a single dose of 0.1 ug/g of body weight, or isotype antibodies was injected as an intravenous (I.V) injection 10 min before the irradiation. Mice were irradiated in a leg restraint box, which meant that only their right tumor-bearing (primary tumors) thigh received a local dose of radiation [13] (Figure S1). The tumor volumes were compared between the mice that received different treatments, whereas the tumor folds, which are the ratio of tumor volume after a specific day of radiation to the tumor volume on the day of radiation and represent the tumor growth rate, were calculated and compared between primary and secondary tumors.

### Analysing mouse tumor apoptosis and infiltrated cytotoxic T cells and dendritic cells

After 6 days of treatment, mice were sacrificed by cervical spine dislocation and tumors were extracted. To obtain a single-cell suspension, a gentle MACS desiccator and a murine tumor dissociation kit (Miltenyi Biotec) were used according to previous studies [26, 27]. For analysis of tumor apoptosis, a single-cell suspension of 1 * 10^6^ cells was prepared from isolated tumor tissue, and 10 mL of this suspension was aliquoted into individual tubes. A total of 5 mL of Annexin Vefluorescein isothiocyanate (FITC, Sigma-Aldrich, catalog number: APOAF-60TST) and 5 mL of propidium iodide (Sigma-Aldrich, catalog number: APOAF-60TST) was added to each tube according to the desired analysis. Cells were incubated in an ice bath for 15 min in the dark, followed by the addition of 400 mL ice-cold 1X binding buffer and gentle mixing. Next, cells were analyzed using an Epics XL flow cytometer (Beckman Coulter, Miami, FL, USA). For the analysis of dendritic cells and T cells, nonspecific binding was blocked first by purified CD16/32. Next, cells were incubated with conjugated antibodies for 30 min at 4 °C, washed with PBS, and centrifuged twice at 300 g for 5 min at 4 °C. A lineage of B cells (CD19), myeloid cells (CD11b), and RBCs (TER-119) was excluded and the gate which was positive for CD45 and CD8 was chosen to analyse cytotoxic T cells (CTLs) by flow cytometry. To analyse the DCs, a lineage of B cells (CD19), T cells (CD3), and RBCs (TER-119) was excluded and the gate, which was positive for CD45, CD11b and CD11C was chosen. CD45 Brilliant violent 605 (anti mouse), CD8 PE/Cy7 (anti mouse), CD11b FITC (anti mouse) and CD11C APC/Fire750 (anti mouse) were from Biolegend.

### Statistical analysis

All statistical analyses were performed using SPSS 14.0 statistical software. p value < 0.05 was considered statistically significant. Survival fraction in 2 Gy (SF2) = number of colonies after 2 Gy radiation /number of colonies in non-irradiated cells. ANOVA was used to calculate the statistical significance between two groups at a given radiation dose. graphics were done using GraphPad Prism 9 software.

## Results

### Anti-CD95 antibodies boost apoptosis after radiation

**Fig. 1:**
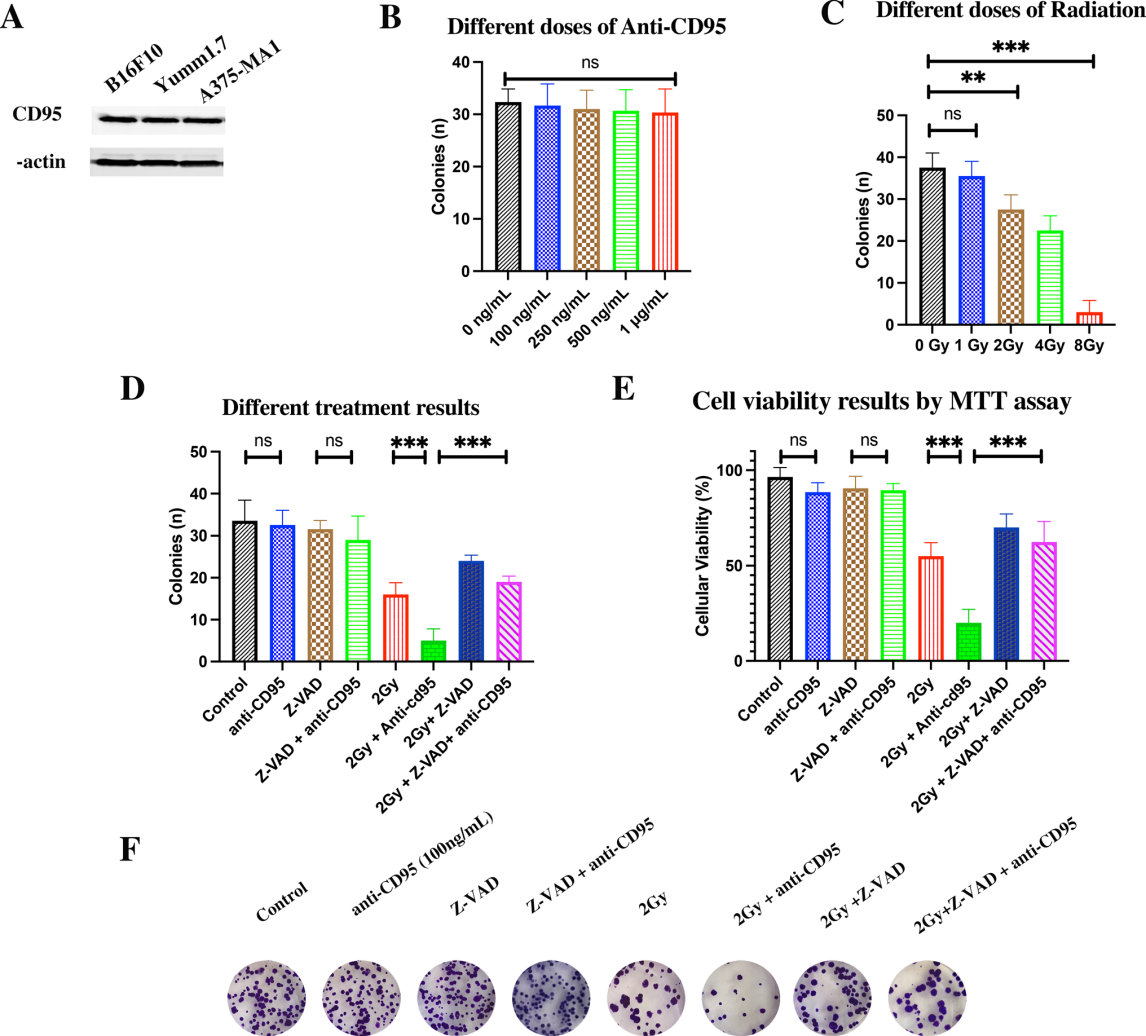
The combination of radiation and anti-CD95 mAb induces apoptosis on B16F10. **A**: CD95 expression in cell lines; **B**: The Anti-CD95 drug dose test on B16F10; **C**: Radiation dose-dependent test on B16F10; **D**: The anti-tumor effect of anti-CD95 plus radiation on B16F10; **E**: The MTT results of anti-CD95 plus radiation on B16F10; **F**: Colony Assay of anti-CD95 plus radiation on B16F10; The data given are the mean values ± S.D. Statistical significance between non-irradiated and irradiated cells was determined by analysis of variance (one way-ANOVA) followed by a Bonferroni’s selected comparisons test. *:p < 0.05, **:p < 0.01, ***:p < 0.001

The expression of CD95 was examined in all cell lines by immunoblotting (Fig. 1A and supplemental Fig S6). The data clearly show that B16F10, YUMM 1.7, and A375-MA1 cells had CD95 expression, which consistent with previous findings [28–30]. Radiation was applied on B16F10, YUMM 1.7, and A375-MA1 cells to determine the dose-dependency of cell death of cells upon radiation. Cells were seeded in 6-well plates and irradiated with different doses (0, 1, 2, 4, and 8 Gy). After 7 days, colonies were counted and the results revealed a significant dose-dependent decrease in colony-forming units compared to non-irradiated cells (control), i.e., 0 Gy. However, treating the cell lines only with different concentrations of anti-CD95 (100 ng/mL, 250 ng/mL, 500 ng/mL, and 1 µg/mL) showed, when colonies were counted 7 days after treatment, no restriction in cell proliferation compared with untreated cells (0 ng/mL) (Fig. 1B, supplemental Fig S3A, and supplemental Fig S4A). Adding anti-CD95 (100 ng/mL) with 2 Gy radiation increased the degree of cell death, which was represented by fewer colonies than treatment with only radiation in B16F10, YUMM 1.7, and A375-MA1 cells (Fig. 1C, supplemental Figure S3B, and supplemental Fig S4B). To further confirm the involvement of apoptosis in the antitumor activity of anti-CD95 plus radiation treatment, cells were treated with Z-VAD-FMK (Z-VAD, 50 µM), a pan-caspase inhibitor, 4 h prior to exposure to anti-CD95 plus radiation. The results showed that cell survival was significantly reduced by anti-CD95 plus radiation treatment, whereas treatment with Z-VAD alone or Z-VAD + anti-CD95 did not affect cell survival. Nonetheless, Z-VAD reverted the anticancer effect of anti-CD95 plus radiation (Fig. 1D, F, supplemental Figure S3C and supplemental Fig S4C). These results were confirmed by MTT assay (Fig. 1E, supplemental Figure S3D, and supplemental Figure S4D). We next verified the induction of CD95 expression upon radiation of tumor cells, we found out that CD95 expression in melanoma cells were increased at 6, and 24 h after 2 Gy and 4 Gy radiation (Figure S2). Our findings show that CD95 expression in melanoma cells is induced after radiation, which may explain why radiated tumor cells are more sensitive to anti-CD95 treatment.

**Fig. 2:**
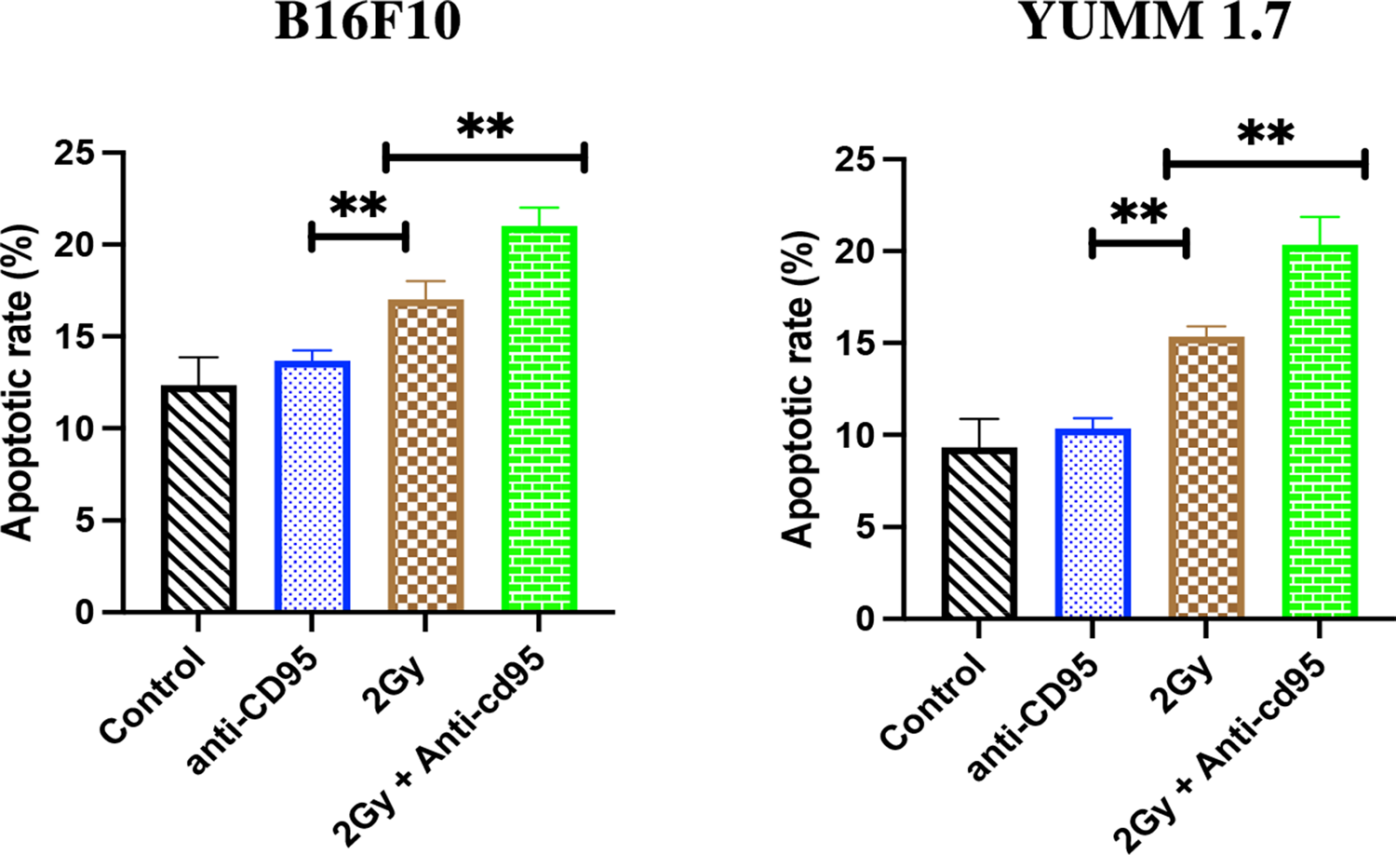
Quantitative analysis of the percentage of apoptosis in mouse xenografts in various treatment groups; Statistical significance was determined by analysis of variance (two-way ANOVA) followed by a Bonferroni’s selected comparisons test. *:p < 0.05, **:p < 0.01, ***:p < 0.001

To be closer to clinical applications, the effect of fractionated irradiation plus anti-CD95 was tested. B16F10 and YUMM 1.7 cells received 3 rounds of 2 Gy radiation plus anti-CD95 treatment. Supplement Table S1 showed that compared with 2 Gy alone, 3 rounds of 2 Gy radiation plus anti-CD95 further reduced cell survival. To further determine the effect of anti-CD95 plus radiation on the induction of apoptosis in melanoma, the apoptosis rates in mouse tumor tissue were examined (Fig. 2). Our data showed that anti-CD95 alone had no effect on controlling melanoma in vitro and in vivo. However, combining anti-CD95 with radiation boosts the effectiveness of radiation and achieves regression in melanoma duplication.

### Targeting CD95 enhances tumor regression in both primary and secondary tumors after radiation

**Fig. 3:**
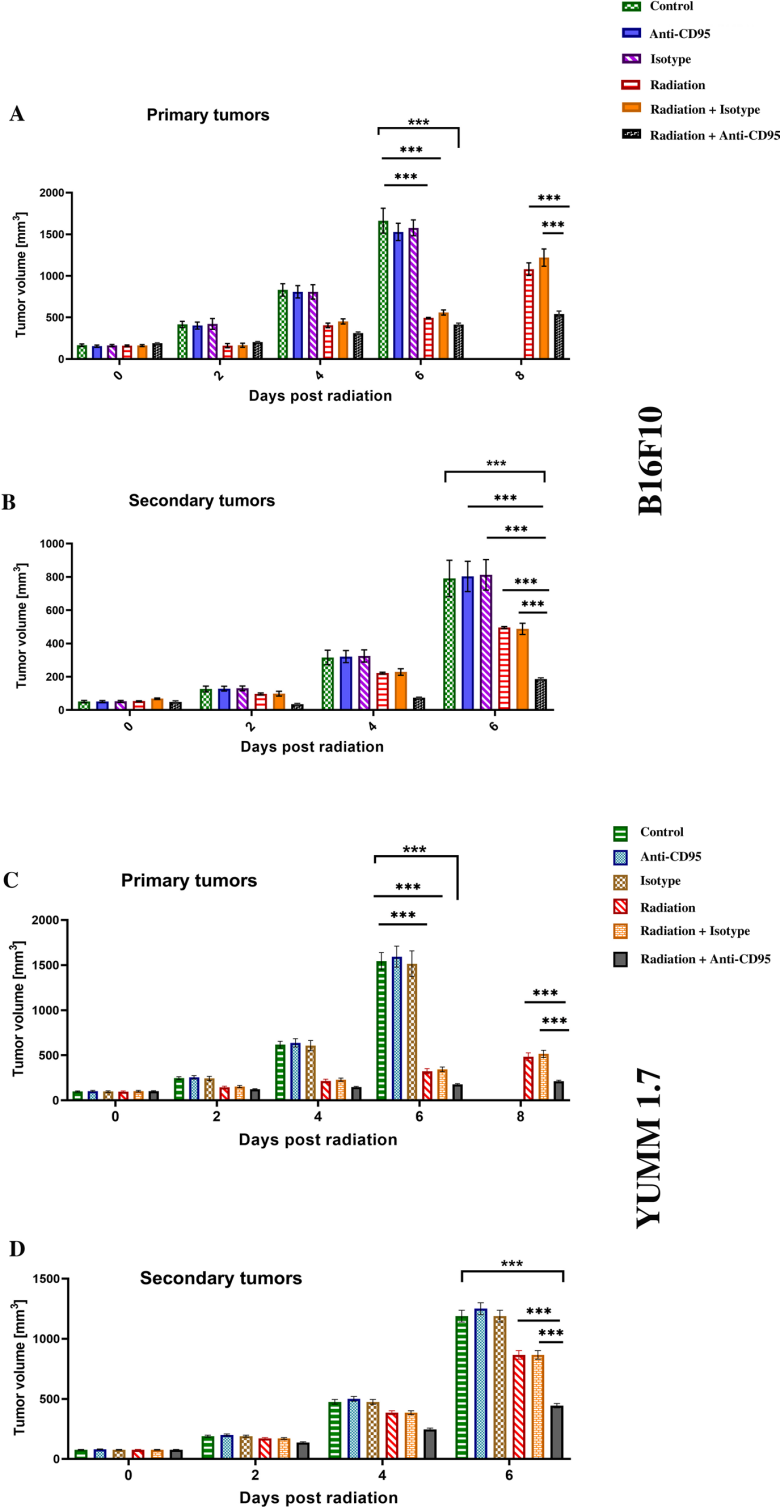
Targeting CD95 enhances tumor regression in both primary and secondary tumors after radiation. **A**: Targeting CD95 enhances B16F10 tumor regression in primary tumor; **B** Targeting CD95 enhances B16F10 tumor regression in secondary tumor after radiation; **C**: Targeting CD95 enhances YUMM 1.7 tumor regression in primary tumor; **D**: Targeting CD95 enhances YUMM 1.7 tumor regression in secondary tumor after radiation; Statistical significance was determined by analysis of variance (two-way ANOVA) followed by a Bonferroni’s selected comparisons test. *:p < 0.05, **:p < 0.01, ***:p < 0.001

**Fig. 4:**
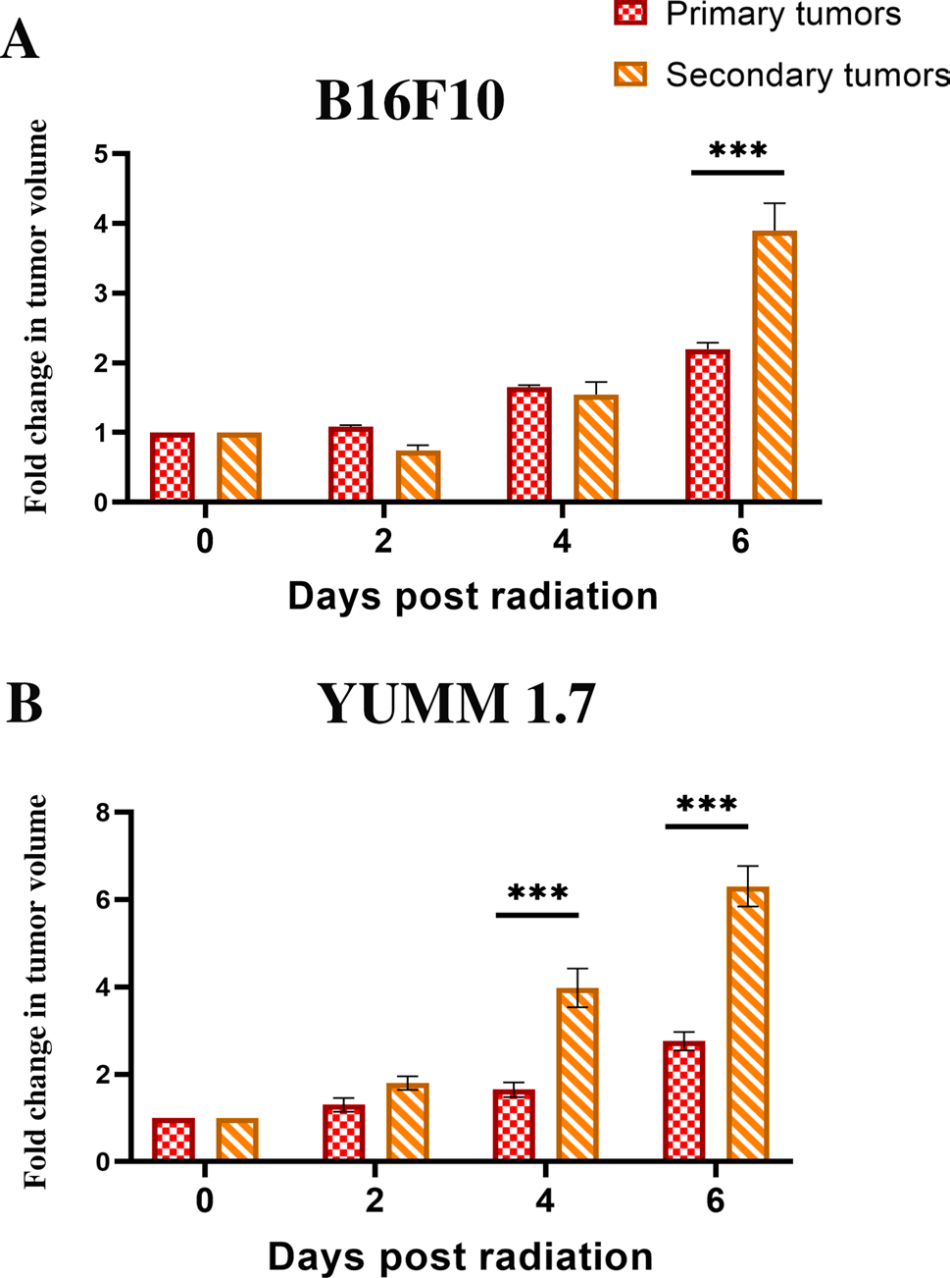
Anti-CD95 mAb plus radiation causes abscopal effect. **A**: YUMM 1.7 Tumor fold in both primary and secondary tumors after radiation; **B**: B16F10 1.7 Tumor fold in both primary and secondary tumors after radiation. Statistical significance in tumor fold, which is the ratio of tumor size at the day of treatment to the tumor size at the endpoint, between the different groups was determined by analysis of variance (two-way ANOVA) followed by a Bonferroni’s selected comparisons test. *:p < 0.05, **:p < 0.01, ***:p < 0.001

Targeting CD95 on melanoma cells hinders tumor growth after radiation in a more efficient manner compared with radiation alone. 1 × 10^5^ B16F10 or YUMM 1.7 cells were subcutaneously injected in a total volume of 50 µl in the lower limbs. Mice with tumors (≈ 100 mm^3^) were classified into a control group that did not receive treatment or a group that received a single administration of anti-CD95 (0.1 µg/g), isotype antibodies (0.1 µg/g), radiation, radiation plus isotype antibodies, or radiation plus anti-CD95 treatment. Primary tumors received local radiation in a single dose of 10 Gy, 10 days after implantation, and antibodies were injected intravenously 10 min prior to the radiation. Primary tumors were measured every 2 days using a digital caliper and the tumor volume was calculated by the equation (Tumor size=(length)*(width)^2^/2). Our data showed that primary tumors were reduced in size among the mice receiving the combination treatment, thereby proving that anti-CD95 was efficient in controlling tumor development under radiation (Fig. 3A, B). Moreover, secondary tumors were measured every 2 days using a digital caliper and the tumor volume was calculated as above. The data showed a slower growth rate in both primary and secondary tumors that received the combination treatment (Fig. 3A, B), which confirmed the effect of anti-CD95 on inducing the abscopal effect. However, primary tumors exhibited a stronger response to treatment compared with secondary tumors (Fig. 4).

### Anti-CD95 combined with radiation induced the recruitment of CTLs and DCs in the tumor microenvironment

**Fig. 5:**
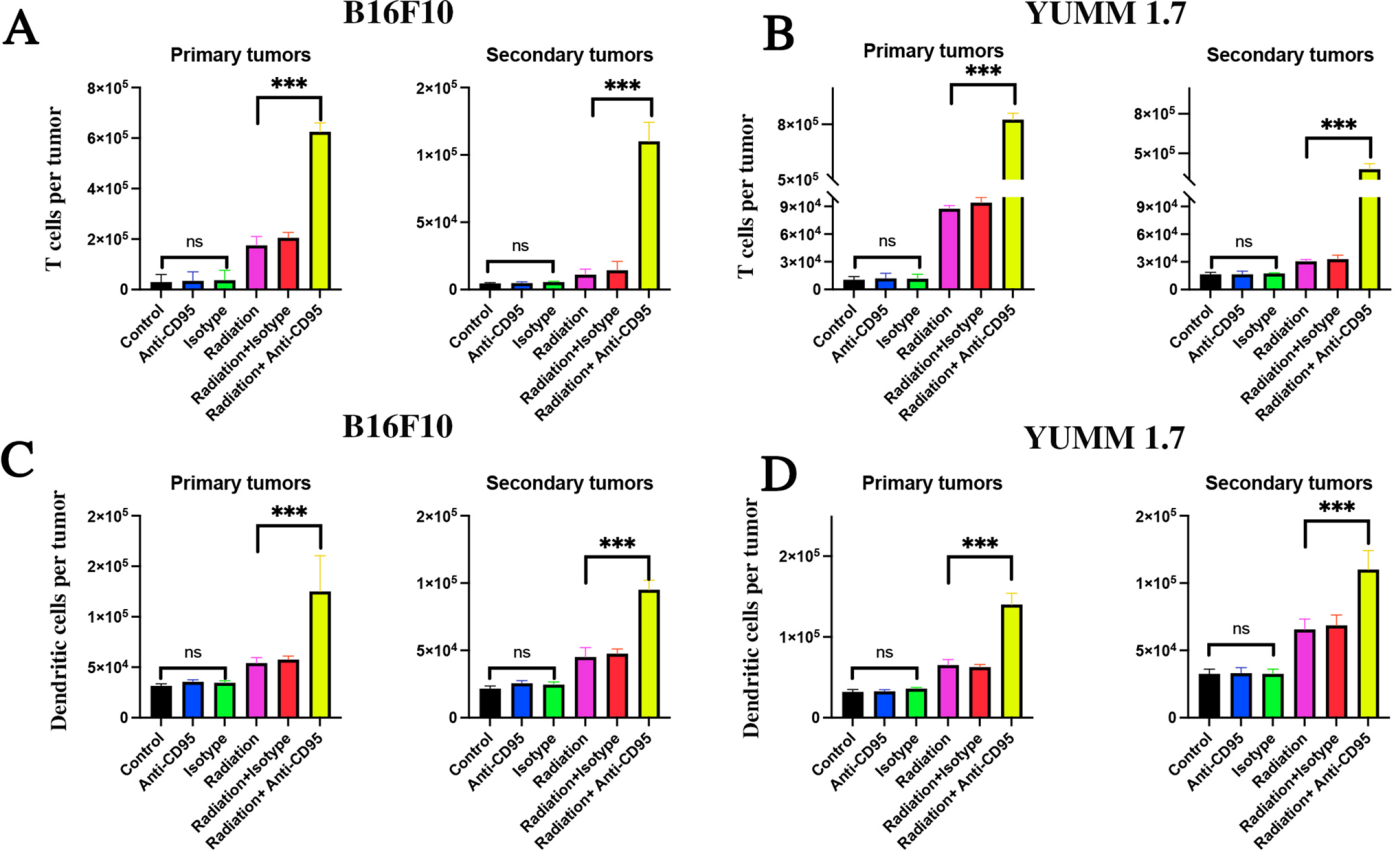
Anti-CD95 mAb plus radiation induced recruitment of CTLs and DCs in Tumor microenvironment. **A**: Anti-CD95 mAb plus radiation treatment promote the infiltration of cytotoxic T cells in both primary and secondary B16F10 tumors; **B**: Anti-CD95 mAb plus radiation treatment promote the infiltration of cytotoxic T cells in both primary and secondary YUMM 1.7 tumors; **C**: Anti-CD95 mAb plus radiation treatment promote the infiltration of dendritic cells in both primary and secondary B16F10 tumors; **D**: Anti-CD95 mAb plus radiation treatment promote the infiltration of dendritic cells in both primary and secondary YUMM 1.7 tumors; Statistical significance between the studied groups was determined by analysis of variance (one-way ANOVA) followed by Bonferroni’s selected comparisons test. ***:p < 0.001

To examine if the abscopal effect in secondary tumors in mice that received combination treatment was induced by the immune response, we studied the infiltration of cytotoxic T cells (CTLs) (Fig. 5A, B) as well as dendritic cells (DCs) (Fig. 5C, D) in both primary and secondary tumors. The extraction of primary and secondary tumors in B16F10 and YUMM 1.7 cells was performed 6 days after multiple treatments with single anti-CD95, isotypes antibodies, 10 Gy radiation, radiation and isotype antibodies, and radiation plus anti-CD95 treatment. Our data showed that without radiation, there was no difference in infiltration of CTLs and DCs between control, anti-CD95, and isotypes groups. The infiltration of CTLs and DCs increased after RT, and the highest degree of infiltration of CTLs and DCs was found in the radiation plus anti-CD95 group, and was observed in both primary and secondary tumors. Thus, the data indicated a role of anti-CD95 in boosting the immune response upon radiation.

## Discussion

Melanoma is a risky type of cutaneous cancer that stems from melanocytes. Representative therapies involve surgical resection at the tumor site in stages 1 and 2, or at the neighboring lymph nodes in stage 3. Because of the spread of the tumor to distant organs, immunotherapy is often performed in stage 4, such as BRAF and MEK inhibitors. But the prognosis of metastatic melanoma patients is still very poor. Current clinical evidence suggests that radiotherapy combined with immunotherapeutic agents could enhance the abscopal effect in metastatic melanoma treatment [31]. However, the precise mechanism of the abscopal effect is still not completely understood in melanoma. In our study, we investigated a potential abscopal effect of combination anti-CD95 and radiotherapy in melanoma. Anti-CD95 plus radiation treatment (combination treatment), single anti-CD95 and single radiation treatment were firstly tested in 3 melanoma cell lines (B16F10, YUMM 1.7, and A375-MA1) in vitro by the colony-formation assay. Our result showed that this combination treatment had a higher apoptosis rate, compared with single anti-CD95 or single radiation treatment. Next, we verified this abscopal effect with the melanoma mouse model. B16F10 or YUMM 1.7 cells were injected in lower limbs of C57BL/6 mice, then different therapies such as single anti-CD95, single radiation or combination treatment were tested in our melanoma mouse model. The data showed that radiating tumors on the right lower limb (primary tumors) and saving tumors of the left part (secondary tumors) caused the growth of primary and secondary tumors to significantly regress in mouse that received combination treatment, thereby proving the abscopal effect. In our experiments, the extraction of primary and secondary tumors proceeded 6 days after treatment, followed by processing as mentioned above to study the infiltrated CTLs and DCs, the main cells of tumor immunotoxicity. Higher infiltration was observed in combination treatment group, compared with other groups. However, the immune response responsible for secondary tumor rejection was not proven to be tumor specific. Based on our observation and previous finding from other groups, we are confident to state that T cell activation is required for the antitumor effect induced by the combination therapy [3, 24]. Previous studies demonstrated that tumor-specific effector CD8 + T cells are required for radiotherapy-induced mobilization of tumor-specific immunity [32], while in our mouse experiments, we found out CD8 + T cells and DCs increased in the tumor treated with combination therapy. The possible mechanism of combination therapy on the immune cells would be as follows: Firstly, CD95 can further enhance the effect of radiation on the tumor-immune cell interaction, such as upregulation of tumor antigens, DCs activation and further T cell activation. Recent findings revealed that extracellular vesicles (EVs) released from tumor cells after radiation are largely immunostimulatory, and combination treatment might also enhance the effect of those EVs. Besides, low doses of CD95 agonists increases primary T cell activation, which leads to more activated tumor specific T cells in mice controlling the growth of both primary and secondary tumors [33, 34]. In addition, CD95L is often expressed on melanoma cells and the interaction between CD95L and CD95 on T cells induces T cell apoptosis. Thus, CD95 antibodies blocked possible CD95L_CD95 interaction and preserved T cells in the tumor. Furthermore, radiation and anti-CD95 treatment may stimulate the release and presentation of tumor antigens to antigen-presenting cells (APCs), such as dendritic cells (DCs), and enhance their cross-presentation to cytotoxic T cells (CTLs), leading to the activation of CTLs and their infiltration into the tumor microenvironment. Lastly, the combination therapy may increase the infiltration of CTLs and DCs into the tumor microenvironment, as suggested by higher degrees of infiltrating CTLs and DCs detected in the combination group compared to the other groups.

Based on our experimental data, we can conclude that the effect of combination therapy is synergistic. In our in vitro experiments, CD95 antibody treatment alone had only minor effect on cell proliferation or apoptosis in melanoma cells, while the combination with radiation almost eliminated all the melanoma cells. Induced CD95 expression by radiation in melanoma cells can explain the combination effect. In vivo experiments, the synergistic effect is manifested by tumor volume of both primary and secondary tumors after combination therapy. It will be fascinating to see how clinical testing of anti-CD95 agonists in cancer. Unfortunately, very few of anti-CD95 agonists have reached clinical trials. But we are looking forward to the results from Phase I clinical trial (NCT02588419) and Phase I/II trial (NCT02277197).

In summary, our experiment results support the immune hypothesis for the abscopal effect in melanoma, and also illustrate the potential of combining radiotherapy and immunotherapy in the treatment of melanoma.

## Electronic supplementary material

**Supplementary file 1**

## Data Availability

The datasets generated and/or analyzed during the current study are not publicly available due to the fact that there are still some related experiments in progress in our group but are available from the corresponding author on reasonable request.
