# Supplementary material for: The abscopal effect of anti-CD95 and radiotherapy in melanoma
Source: Discov Oncol. 2023 May 16;14:68. doi: 10.1007/s12672-023-00682-7 (PMC10188694; doi:10.1007/s12672-023-00682-7)
Supplement: Supplementary file 1 [file 12672_2023_682_MOESM1_ESM.docx]

**Supplementary Materials**

**Title: The abscopal effect of anti-CD95 and radiotherapy in melanoma**

**
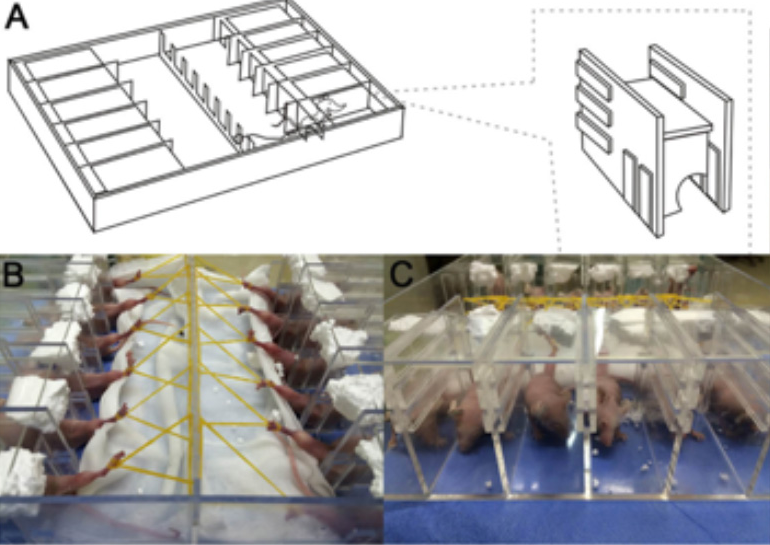
**

**Figure S1:** leg restraint box^1^. ^1^mice were fixed in a small room, used a fine line to pull out theirs right thigh from a hole and then fixed the line to a pillar. Then their right thigh received local radiation dose; A: The sketches of leg restraint box; B and C: Practical application of leg restraint box.





**Figure S2:** Immunoblotting results of CD95 expression after radiation


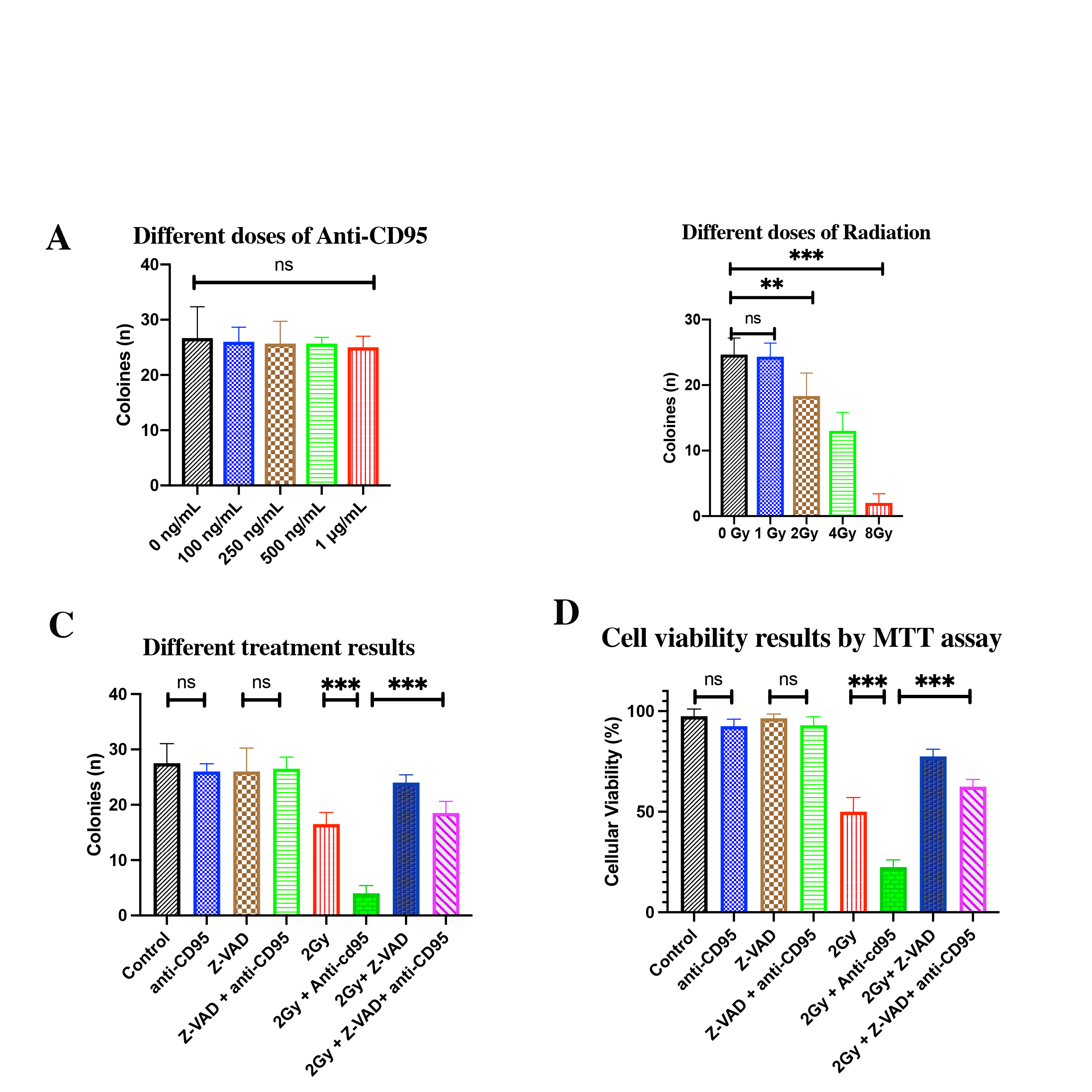


**Figure S3:** The combination of radiation and anti-CD95 mAb induces apoptosis on YUMM 1.7.A: The Anti-CD95 drug dose test on YUMM 1.7; B: Radiation dose-dependent test on YUMM 1.7; C: The anti-tumor effect of anti-CD95 plus radiation on YUMM 1.7; D: The Apoptotic effect of anti-CD95 plus radiation on YUMM 1.7;The data given are the mean values ± S.D. Statistical significance between non-irradiated and irradiated cells was determined by analysis of variance (one way-ANOVA) followed by a Bonferroni’s selected comparisons test. *:p<0.05, **:p<0.01, ***:p<0.001

**
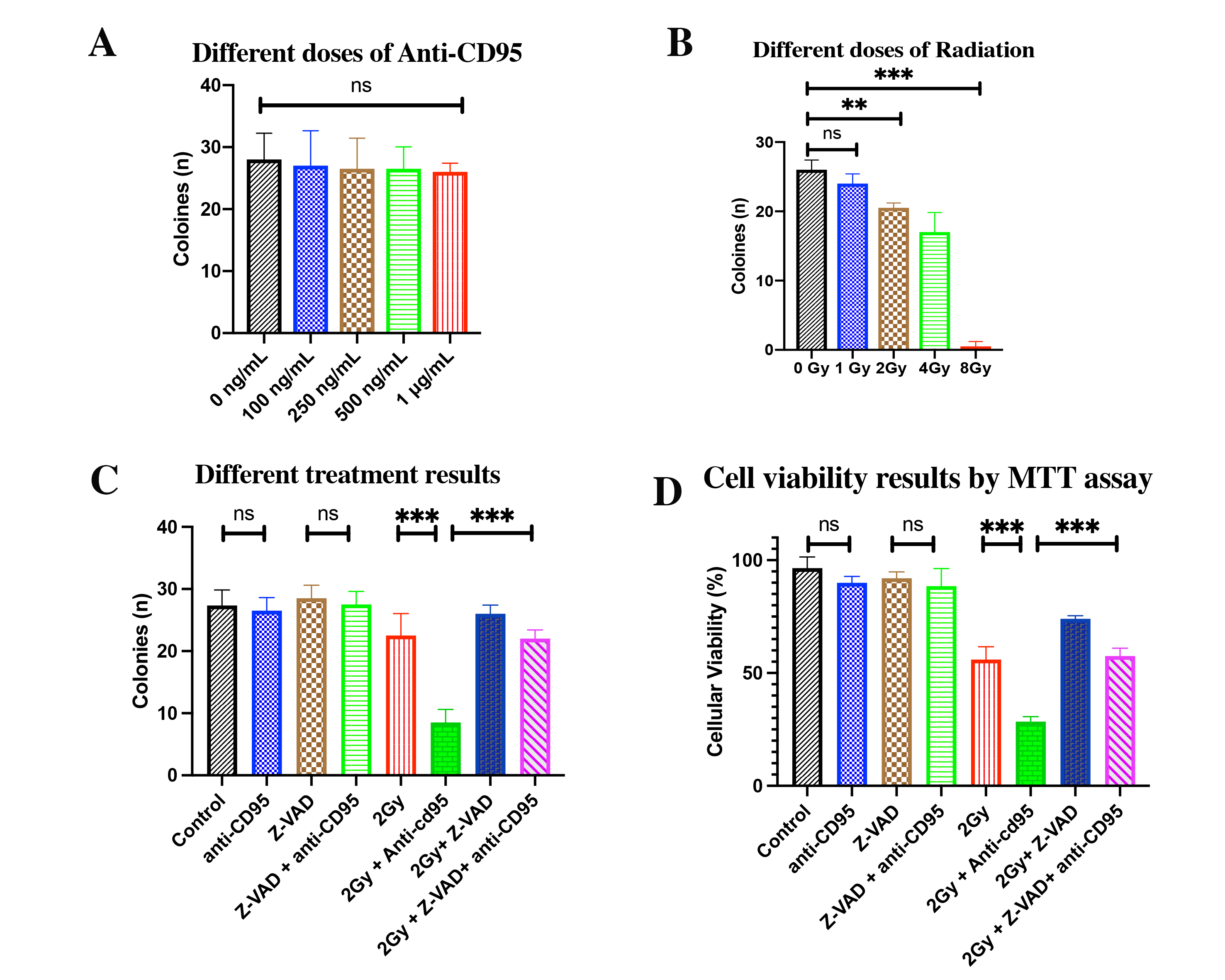
**

**Figure S4:** The combination of radiation and anti-CD95 mAb induces apoptosis on A375-MA1.A: The Anti-CD95 drug dose test on A375-MA1; B: Radiation dose-dependent test on A375-MA1; C: The anti-tumor effect of anti-CD95 plus radiation on A375-MA1; D: The Apoptotic effect of anti-CD95 plus radiation on A375-MA1; The data given are the mean values ± S.D. Statistical significance between non-irradiated and irradiated cells was determined by analysis of variance (one way-ANOVA) followed by a Bonferroni’s selected comparisons test. *:p<0.05, **:p<0.01, ***:p<0.001.


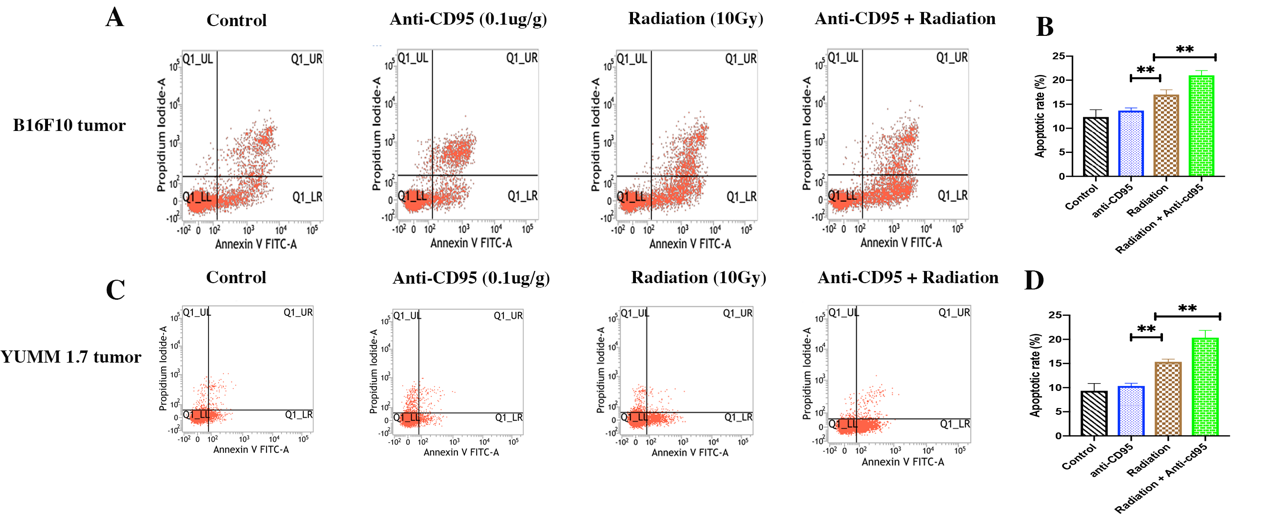


**Figure S5:** Anti-CD95 mAb plus radiation causes apoptosis in mice model. A: Apoptosis rates of B16F10 tumor tissue in different groups were performed by flow cytometry analysis; B: Quantitative analysis of the percentage of apoptosis in B16F10 mouse xenografts in various treatment groups; C: Apoptosis rates of YUMM 1.7 tumor tissue in different groups were performed by flow cytometry analysis; D: Quantitative analysis of the percentage of apoptosis in YUMM 1.7 mouse xenografts in various treatment groups; Statistical significance was determined by analysis of variance (two-way ANOVA) followed by a Bonferroni’s selected comparisons test. P-values (p≤0.01) were indicated by an asterisk**. n =3


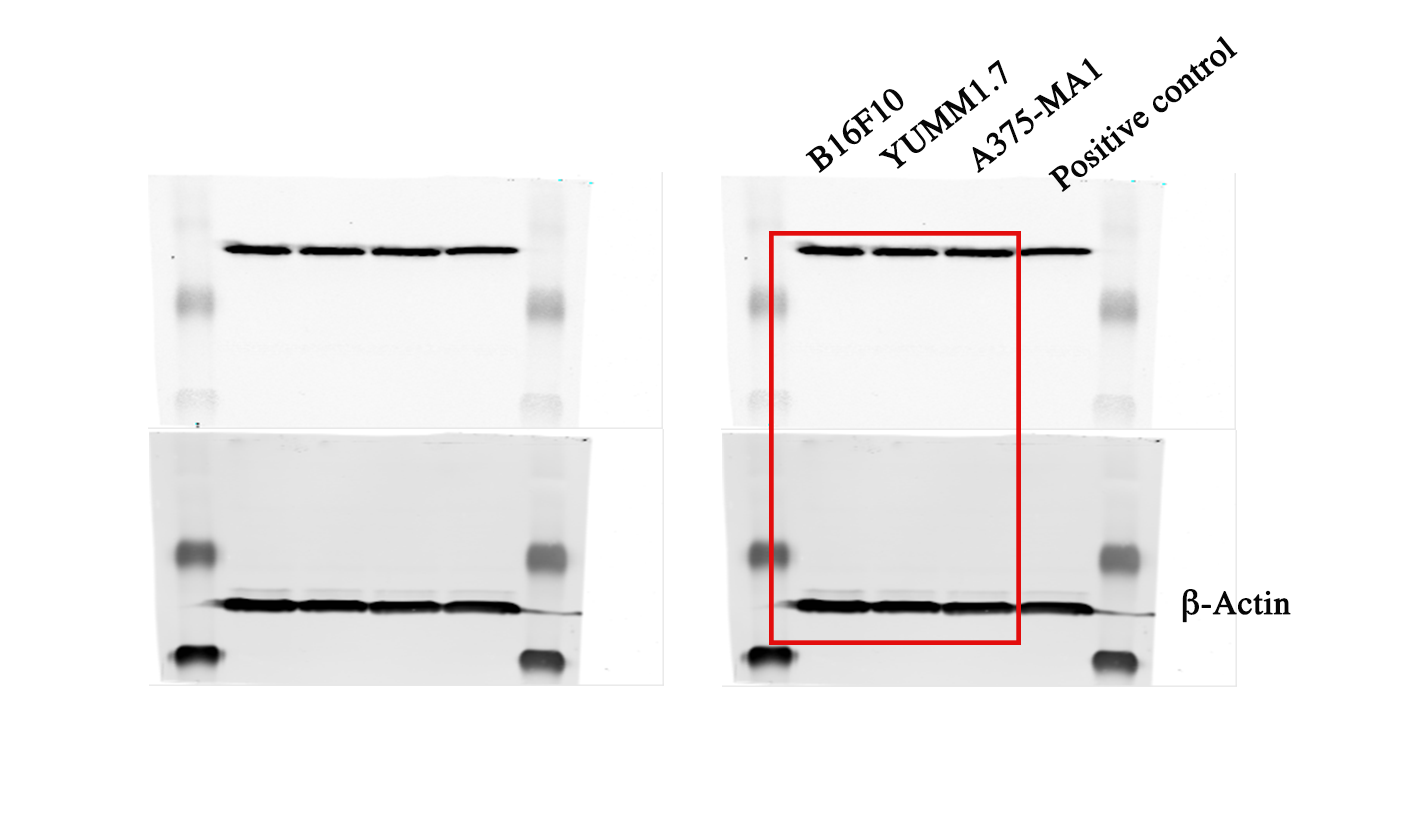
**Figure S6:** Immunoblotting results of CD95 in cell lines

| **B16F10** | **SF2** | **P** |
| --- | --- | --- |
| 2Gy | 0.37 ± 0.04 | 0.023 |
| 2Gy + Anti-CD95 | 0.26 ± 0.032 |  |
| 3 * 2Gy | 0.17 ± 0.023 | 0.004 |
| 3 * 2Gy + Anti-CD95 | 0.08 ± 0.015 |  |
| **YUMM 1.7** | **SF2** | **P** |
| 2Gy | 0.29 ± 0.018 | 0.01 |
| 2Gy + Anti-CD95 | 0.17 ± 0.04 |  |
| 3 * 2Gy | 0.13 ± 0.015 | 0.001 |
| 3 * 2Gy + Anti-CD95 | 0.02 ± 0.012 |  |

**sTable1:** The effect of fractionated irradiation plus an-CD95

SF2: survival fraction in 2Gy. P: ANOVA was used to calculate the statistical significance between groups, n =3.
